# Chronic gastric volvulus—Case report of an uncommon diagnosis

**DOI:** 10.1016/j.ijscr.2019.11.029

**Published:** 2019-11-27

**Authors:** M.J. Jervis, R. Candeias, E. Duro, L.G. Pereira, F. Caratão

**Affiliations:** Local Health Unit of the Lower Alentejo, General Surgery Department. Rua Dr. António Ferreira Covas Lima, 7801-849, Beja, Portugal

**Keywords:** Gastric volvulus, Hiatal hernia, Nissen fundoplication, Case report

## Abstract

**Introduction:**

Gastric volvulus is an uncommon condition that results from the torsion of the stomach upon its axis. Although rare, it can present as an acute medical emergency, therefore its diagnosis should not be overlooked. In this article we aim to present a case of a patient with chronic gastric volvulus operated in our hospital. We also aim to discuss this condition.

**Presentation of case:**

We report the case of a 61  year old, healthy male, referred to surgical consultation complaining of epigastric discomfort and postprandial fullness. The diagnostic workup showed a mesentero-axial volvulus of the stomach. He was operated electively: reduction of volvulus, Nissen fundoplication and gastropexy was performed, with resolution of symptoms.

**Discussion:**

There are several predisposing factors for the development of a gastric volvulus. It is classified according to the axis upon the rotation of the stomach occurs. The treatment of gastric volvulus may differ in the acute or chronic setting.

**Conclusion:**

Diagnosis of a chronic gastric volvulus requires a high index of suspicion. Surgical repair should be done to prevent an acute complication.

## Highlights

• Chronic gastric volvulus is an uncommon condition.
• The diagnosis of a chronic gastric volvulus requires a high index of suspicion.
• Surgical repair should be done to prevent an acute complication, associated with higher morbidity and mortality.

## 1 Introduction

Gastric volvulus is an uncommon condition that results from the torsion of the stomach upon its axis [[1], [2], [3]]. Its true incidence is unknown [4], with 15 % of cases occurring in children under 1 year of age with congenital diaphragmatic defects [2,5]. In adults it occurs more frequently in the fifth decade of life, with similar prevalence in both genders [2,4,5].

Chronic gastric volvulus may be asymptomatic, or present with non-specific symptoms such as dysphagia, heartburn, epigastric discomfort or fullness, and bloating, usually postprandial [1,2,6].

Its typical acute presentation is described by the Borchardt’s triad [2,6], characterized by sudden-onset intense epigastric pain, inability to pass a nasogastric tube and retching without productive emesis.

When it presents in its acute form it can become a surgical emergency [3] with increased morbidity and mortality [1].

In this article, we present a case of a patient with chronic gastric volvulus operated in our hospital. This case report is compliant with the SCARE guidelines [10].

## 2 Presentation of case

We present a case of a 61 year old, healthy male, that was referred to surgical consultation complaining of epigastric discomfort and postprandial fullness that alleviated with flexure of the torso, for the past 5 years. He presented no alterations in the physical exam. He had no previous surgeries or known medical conditions.

Diagnostic workup was performed with upper endoscopy, thoraco-abdomino-pelvic CT scan and barium upper gastrointestinal (UGI) radiogram.

The endoscopy described an alteration of the anatomy of the stomach with a highly located antrum, parallel to the cardia with an associated paraesophageal hernia.

**Fig. 1:**
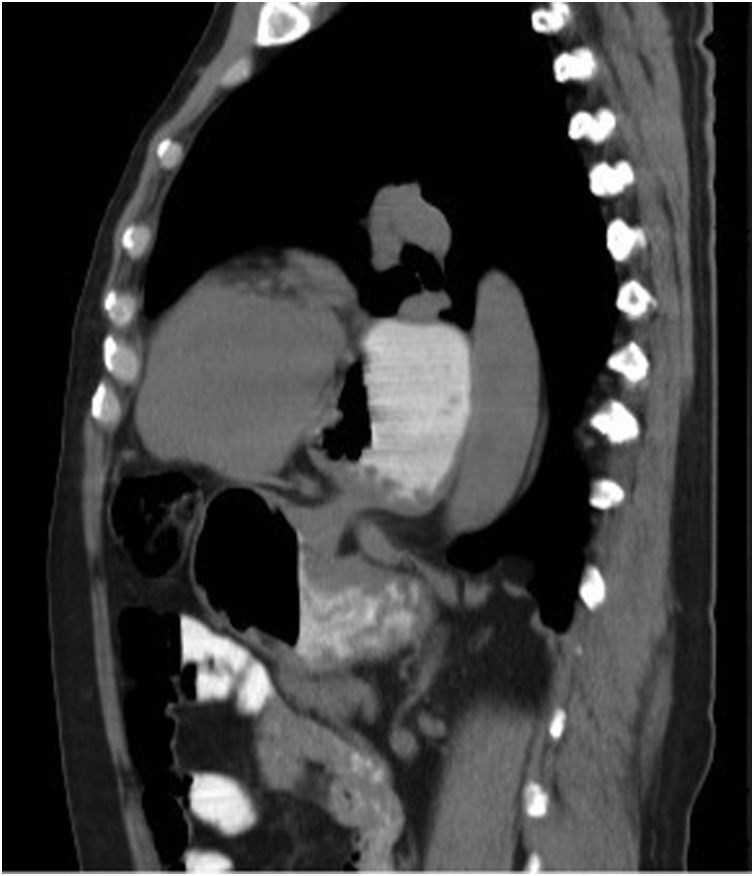
Thoraco-abdomino-pelvic CT scan showing a volumous paraesophageal gastric herniation.

The CT scan (Fig. 1) mentioned a volumous paraesophageal gastric herniation, with the stomach located in the infero-posterior mediastinum, pushing the heart anteriorly.

**Fig. 2:**
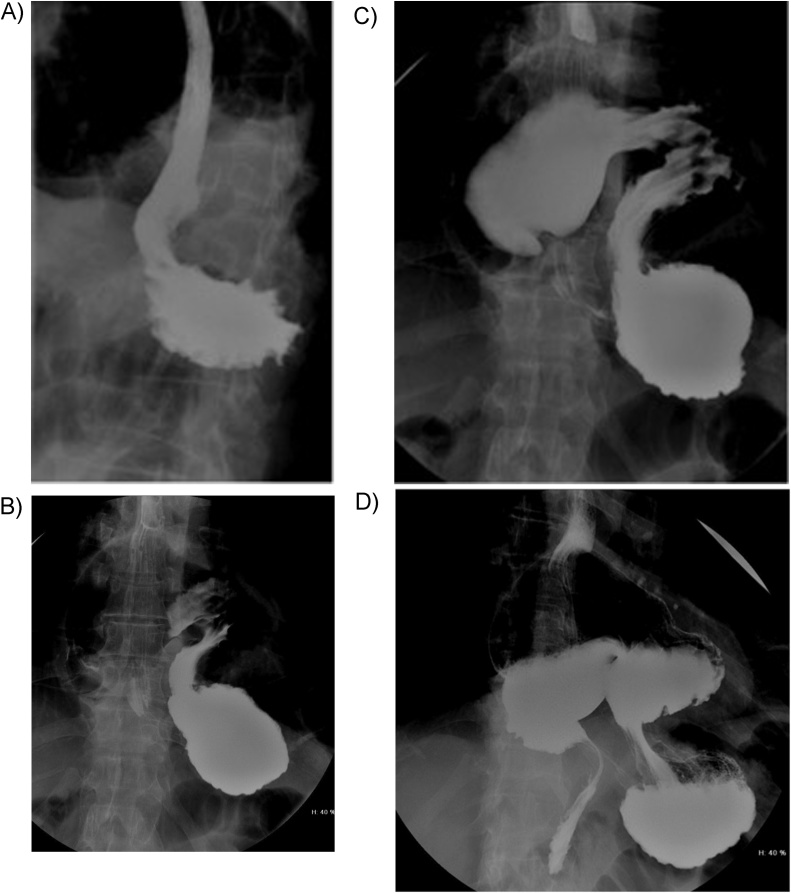
Barium UGI radiogram showing: A - the oesophagus and gastric fundus; B - the gastric fundus; C - the gastric fundus, the gastric body in an intrathoracic location and the antrum in a superior location; D - the gastric fundus, the gastric corpus in an intrathoracic location and the antrum in a superior location with progression of contrast to the duodenum.

The barium UGI radiogram (Fig. 2A–D) showed a rotation of the stomach upon its mesenteric axis, with the gastric fundus located inferiorly to the gastric antrum – mesentero-axial volvulus.

The patient was proposed to elective surgery. Intra-operatively we found a volumous hiatal hernia containing part of the body of the stomach with rotation upon its mesentero-axial axis. Detorsion of the volvulus, reduction of the hernia, excision of the hernia sac, Nissen fundoplication and gastropexy was performed. There were no complications associated to the procedure. The patient had transient hiccups after surgery that resolved spontaneously after several weeks. He was asymptomatic in the follow-up consultation.

## 3 Discussion

The normal fixation of the stomach is attained with the gastrosplenic, gastrohepatic, gastrolienal and gastrocolic ligaments [1,9]. The distal pexy of the stomach results from the fixation of the duodenum to the retroperitoneum [1,2].

Laxity in these ligamentous attachments can result in a gastric volvulus [2,6]. Other predisposing factors include: elevation of the left hemidiaphragm [2], focal adhesions [2], gastric tumour [2], masses in adjacent organs [2], paraesophageal hernia [2], diaphragm (phrenic nerve) palsy [1,3], traumatic diaphragmatic hernia [1], gastric distension [1,3] and abnormalities of the spleen [3].

In two thirds of cases, the volvulus occurs above the diaphragm in association with a paraesophageal or mixed diaphragmatic hernia. In the other third of cases, volvulus occurs below the diaphragm [2].

The gastric volvulus are classified according to the axis in which the rotation occurs. It can be organo-axial [[1], [2], [3], [4], [5], [6], [7], [8], [9]], mesentero-axial [[1], [2], [3], [4], [5], [6], [7], [8], [9]] or mixed/combined [2,5,8,9].

The organo-axial volvulus occurs when there is a rotation upon the longitudinal axis of the stomach, that is the line between the cardia and the pylorus. The antrum moves superiorly and the intrathoracic stomach is usually located in the right hemithorax.

The mesentero-axial volvulus is a rotation upon the mesenteric axis, along a line connecting the middle of the lesser curvature to the greater curvature, frequently causing a right to left or vice-versa rotation, with a typical “upside-down” appearance [[1], [2], [3],^7^]. The intrathoracic stomach is usually located in the left hemithorax.

A mixed or combined gastric volvulus has also been reported and it occurs when the stomach rotates in its both axis [2,5,8,9] (organo-axial and mesentero-axial).

The diagnosis of a chronic or intermittent gastric volvulus requires a high index of suspicion [6,8] because it causes no specific symptoms.

There are characteristic signs [1] in radiographic studies, such as an intrathoracic stomach with a double air-fluid level in the chest radiograph [3,6].

The gold standard diagnostic test is the upper gastrointestinal barium study, as it identifies the position of the stomach and the degree of rotation [6]. It also permits the identification of a concomitant paraesophageal hernia.

The CT scan shows an intrathoracic stomach, with torsion [3] and it is useful to detect signs of gastric ischemia [1] in the acute setting.

Upper gastrointestinal endoscopy can be both diagnostic and therapeutic [6].

The treatment of gastric volvulus will differ in the acute or chronic setting.

The acute gastric volvulus represents a surgical emergency with 30 % mortality [1,2,9] if gastric necrosis as occurred.

Depending on the severity of presentation and patient comorbidities, endoscopic decompression and temporary detorsion may be considered, if there are no signs of ischemia [2,8]. Gastropexy and repair of the associated diaphragmatic defect should be performed by surgery, although there are reported cases of endoscopic gastropexy with gastrostomy tube placement [2,8].

Treatment of chronic gastric volvulus can be performed electively, it includes reduction of the volvulus, repair of the underlying cause (eg. paraesophageal hernia), fundoplication and anterior abdominal wall gastropexy [4,6].

## 4 Conclusion

Diagnosis of a chronic gastric volvulus requires a high index of suspicion. Surgical repair should be done to prevent an acute complication, associated with higher morbidity and mortality.

## Sources of funding

The authors have declared no funding.

## Ethical approval

This case report is exempt from ethical approval in our institution.

## Consent

Written informed consent was obtained from the patient for publication of this case report and accompanying images. A copy of the written consent is available for review by the Editor-in-Chief of this journal on request.

## Author contribution

All authors have contributed to the publication of this article.

## Registration of research studies

Not applicable.

## Guarantor

JH RICHARD DAVID David ROSIN.

## Provenance and peer review

Not commissioned, externally peer-reviewed.

## Declaration of Competing Interest

The authors declare no conflicts of interest.
